# Steroid administration facilitates recovery in severe refractory Bell’s palsy patients who did not receive steroids in the acute stage: a retrospective study

**DOI:** 10.3389/fneur.2026.1760155

**Published:** 2026-02-11

**Authors:** Sha-Sha Ding, Kai-Hua Song, Ying-Ying Zhang, Fu Xu, Li-Hua Xuan, Shou-Hai Hong

**Affiliations:** 1 Department of Rehabilitation, Tianjin Nankai Hospital, Tianjin Medical University, Tianjin, China; 2 Tianjin Key Laboratory of Acute Abdomen Disease Associated Organ Injury and ITCWM Repair, Tianjin, China; 3 Institute of Integrative Medicine for Acute Abdominal Diseases, Tianjin, China; 4 The First Clinical Medical College, Zhejiang Chinese Medical University, Hangzhou, Zhejiang, China; 5 Children’s Hospital, Zhejiang University School of Medicine, Hangzhou, China; 6 Department of Acupuncture and Moxibustion, The First Affiliated Hospital of Zhejiang Chinese Medical University, Hangzhou, China

**Keywords:** acupuncture, Bell’s palsy, facial nerve, recovery stage, steroids

## Abstract

**Background:**

In the present study, we aimed to evaluate the efficacy of steroid treatment on patients with severe refractory Bell’s palsy in the recovery stage who had not been treated with steroids in the acute stage.

**Methods:**

We performed a retrospective analysis of 122 patients with severe refractory Bell’s palsy. These patients had a clinical course of 15–30 days, House-Brackmann (HB) grade ≥ IV, and no history of steroid therapy. Of those, 65 patients who received steroid therapy after hospitalization were assigned to the steroid group and 57 patients who did not receive steroid therapy were assigned to the control group. Both groups received folic acid and mecobalamin, acupuncture, and mirror exercises as basic treatments. The primary outcome was the rate of grade I on the HB Grading Scale. The secondary outcome was the Sunnybrook Facial Grading system. All outcomes were assessed before treatment (baseline), at 2 weeks of steroids treatment (2 weeks), and at 8 weeks after steroids treatment (10 weeks).

**Results:**

The steroid group had a better recovery rate of facial-nerve function than the control group (*p* < 0.05).

**Conclusion:**

The use of steroids facilitate the recovery of severe refractory Bell’s palsy in the recovery stage, who did not receive steroids in the acute stage.

## 1 Introduction

Bell’s palsy is an acute, idiopathic peripheral facial paralysis that presents with sudden unilateral weakness or paralysis of the facial muscles (1). It is the most common cause of peripheral facial paralysis, responsible for 60%–75% of cases. Epidemiological studies have reported that 11.5–53.3 cases occur per 100,000 people each year (2, 3). Inflammation of the facial nerve has been considered to be the cause of Bell’s palsy, but the initiating factor remains unclear (4, 5). Viral infection, vascular ischaemia, autoimmune inflammatory disorders and heredity have been put forward as the underlying causes (4).

Even though the majority of Bell’s palsy patients have a good prognosis, around 16% of them develop permanently reduced function with abnormal innervation (motor synkinesis or autonomic dysfunction), and postparalytic spasms (6). These sequelae are disabling conditions that negatively affect psychosocial well-being and quality of life (7). In fact, a study found that the positive rate of depression was higher in patients with poor recovery of facial paralysis (8).

The recent practice guidelines (1, 9) strongly recommend that steroid therapy should be started as early as possible after symptom onset (within 72 h) to better promote the recovery of facial nerve function and significantly reduce the risk of synkinesis. Research found that with early steroids use, the full recovery rate of facial paralysis could exceed 90% (10). However, some patients still did not receive early steroid treatment due to the substandard treatment or fear of side effects. As a well-known key research center for Bell’s palsy in Zhejiang Province, our institution treats a large number of such patients. This has enabled us to find a specific, hard - to - treat group: those with a clinical course of 15–30 days and HB grade ≥ IV who showed poor recovery even without early steroid treatment. The enhancement of facial nerve in these patients was shown in Magnetic Resonance Imaging (MRI) using gadolinium enhancement, suggesting that facial nerve inflammation still existed. Thus, this study was designed to explore the effect of the steroid in the recovery stage on the severe refractory Bell’s palsy patients who did not receive steroid therapy during the acute stage.

## 2 Materials and methods

We did a retrospective analysis of patients diagnosed with Bell’s palsy at the First Affiliated Hospital of Zhejiang Chinese Medical University between 2015 and 2019. The HB scale was used to evaluate disease severity. Based on the retrospective data, 122 patients met the inclusion criteria. In this analysis, these patients were divided into two cohorts: 65 patients receiving steroid therapy (steroid group) and 57 not receiving it (control group). Both groups receiving concurrent routine treatment.

Bell’s palsy was diagnosed by ruling out all other causes of peripheral facial paralysis. The inclusion criteria were as follows: patients aged 18–65 years who had unilateral facial-nerve weakness without an identifiable cause; patients with 15–30 days history of Bell’s palsy and HB grade ≥ IV (defined as severe refractory facial paralysis); patients who had not received steroids therapy after onset of symptoms; patients who had received MRI with gado-linium enhancement to explore the entire course of the facial nerve after hospitalization. The exclusion criteria were as follows: patients with bilateral paralysis or recurrent facial paralysis; patients with a history of tumors, hepatitis, tuberculosis, gastric ulcer, severe hypertension, uncontrolled diabetes, liver and kidney dysfunction, mental illness, or serious systemic diseases that might affect the treatment; patients who had recently undergone surgery or trauma; pregnant and breastfeeding women.

All patients were given standardized acupuncture treatment in accordance with contemporary Chinese clinical practice guidelines for Bell’s palsy (11). In short, five sessions per week over 4 weeks were conducted by certified acupuncturists. Sterilized stainless steel needles (Φ0.25 mm × 40 mm) were inserted at selected facial and limb acupoints like Yangbai (GB14), Hegu (LI4), and others. For patients without synkinesis, a low-intensity dilatational wave electrical stimulation was applied to specific facial acupoint pairs. If synkinesis was seen, the stimulation was stopped right away, and warm acupuncture was used instead.

In the steroid group, in addition to the routine treatment, the patients also received steroid therapy for 15 days. This included intravenous dexamethasone at a dose of 10 mg/d for 3 days, 5 mg/d for 3 days, and orally prednisone at a dose of 30 mg/d for 3 days, 20 mg/d for 3 days, and 10 mg/d for 3 days. In the control group, the patients did not receive steroids.

Intravenous administration of dexamethasone and acupuncture treatment were provided during the hospitalization. Oral administration of prednisone was continued after discharge and the remaining acupuncture treatment was carried out in the outpatient department. Follow-up visit was conducted in the outpatient department after the treatment was completed.

The primary outcome was the proportion of patients who had grade I on the HB Grading Scale. The grading of facial paralysis was done with the HB Grading Scale, which is based on six grades of score: grade I means normal function, and grade VI means complete paralysis. Patients were required to demonstrate four standard expressions of the face: at rest, raising eyebrows, closing eyes tightly, and showing teeth. The secondary outcome was disability measured by the Sunnybrook Facial Grading System, which is regionally weighted and measures resting symmetry, symmetry of voluntary movements, and synkinesis. The composite score is between 0 (complete facial paralysis) and 100 (normal function). All outcomes were assessed by two independent neurologists who were blinded to the assignment of patient’s treatment groups, based on standardized photographs and video recordings taken at the specified time points. The inter-rater reliability was high (Cohen’s kappa > 0.8 for HB scale). Assessments were performed before treatment (baseline), at 2 weeks of steroid treatment (2 weeks), and at 8 weeks after steroid treatment (10 weeks).

## 3 Statistical analysis

All data were analyzed using the Statistical Package for the Social Sciences (SPSS) version 19.0 (SPSS Inc., Chicago, IL, United States). A *p*-value <0.05 was taken as statistically significant. For patient baseline characteristics, the continuous variable data were analyzed by *t*-test (normally distributed data), and the categorical data were analyzed by chi-square test. For the HB Grading scale and Sunnybrook Facial Grading System scores (which are not normally distributed data), a non-parametric Friedman test was used to examine the effect of time (baseline, 2 weeks, and 10 weeks) for each group separately. Mann–Whitney pairwise comparisons were used for the overall change score for each individual between both groups at the three time points. Chi-square test was used to compare the proportion of patients with grade I on the HB Grading Scale between the two groups.

## 4 Results

Of the 122 patients enrolled, 65 patients were assigned to the steroid group and 57 patients were assigned to the control group. Patient characteristics were summarized in Table 1. The two groups were age- and sex- matched. The average age of the steroid group was 43.97 years old, and that of the control group was 39.81. The male-to-female ratio was 35/30 in the steroid group and 23/34 in the control group. The steroid group and the control group had similar age and sex distributions.

**Table 1:** Epidemiological and clinical characteristics of the study population.

Characteristics	Steroid group (*n* = 65)	Control group (*n* = 57)	*p* value
Gender [*n* (%)]			
Female	35 (53.9)	23 (40.4)	1.4
Male	30 (46.2)	34 (59.7)	1.4
Age (years)			
Mean (SD)	44.0 (16.1)	39.81 (16.2)	0.9
Side (%)			
Right	38 (58.5)	31 (54.4)	0.7
Left	27 (41.4)	26 (45.6)	0.7
Hypertension [*n* (%)]	23 (35.4)	21 (36.8)	0.9
Diabetes mellitus [*n* (%)]	13 (20.0)	16 (28.1)	0.3

Data are presented as mean ± standard deviation or number (%).

The difference in the distributions of left-sided and right-sided facial paralysis between the two groups was not statistically significant: the left-to-right ratio was 38/27 in the steroid group and 31/26 in the control group. The distributions of patients with hypertension or diabetes mellitus in the two groups had no statistical difference.

**Figure 1:**
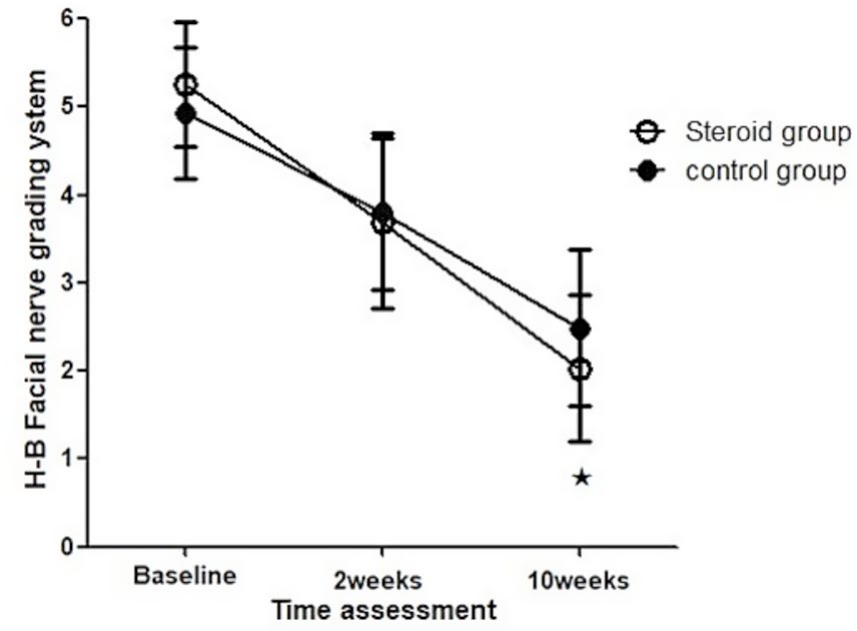
Changes in HB grading scale between both groups at different time points of assessment.

HB scores of the groups were analyzed individually. A non-parametric Friedman test revealed that time had a significant effect on both groups (*p* = 0.000 for each group). Mann–Whitney pairwise comparisons were used to compare the overall change in HB scores between the two groups at various times of assessment. It indicated a significant difference between both groups with greater improvement in the steroid group at 10 weeks (2.5 ± 0.9 vs. 2.03 ± 0.83, *p* = 0.005) (Figure 1).

The proportion of patients with grade I on the HB Grading Scale between groups at baseline and 2 weeks were both 0% (Table 2); however, 7 (12.28%) patients showed grade I in the control group and 19 (29.23%) patients showed grade I in the steroid group at 10 weeks, which showed a significant difference between both groups (*p* = 0.023).

**Table 2:** Changes in the HB grading scale between both groups at different times of assessment.

Group	*n*	Group	Grade I	Grade II	Grade III	Grade IV	Grade V	Grade VI
Control group	57	Baseline	0	0	0	18	25	14
		2 weeks	0	4	16	25	11	1
		10 weeks	7	22	22	5	1	0
Steroid group	65	Baseline	0	0	0	10	28	27
		2 weeks	0	7	22	21	13	1
		10 weeks	19	27	17	2	0	0

**Figure 2:**
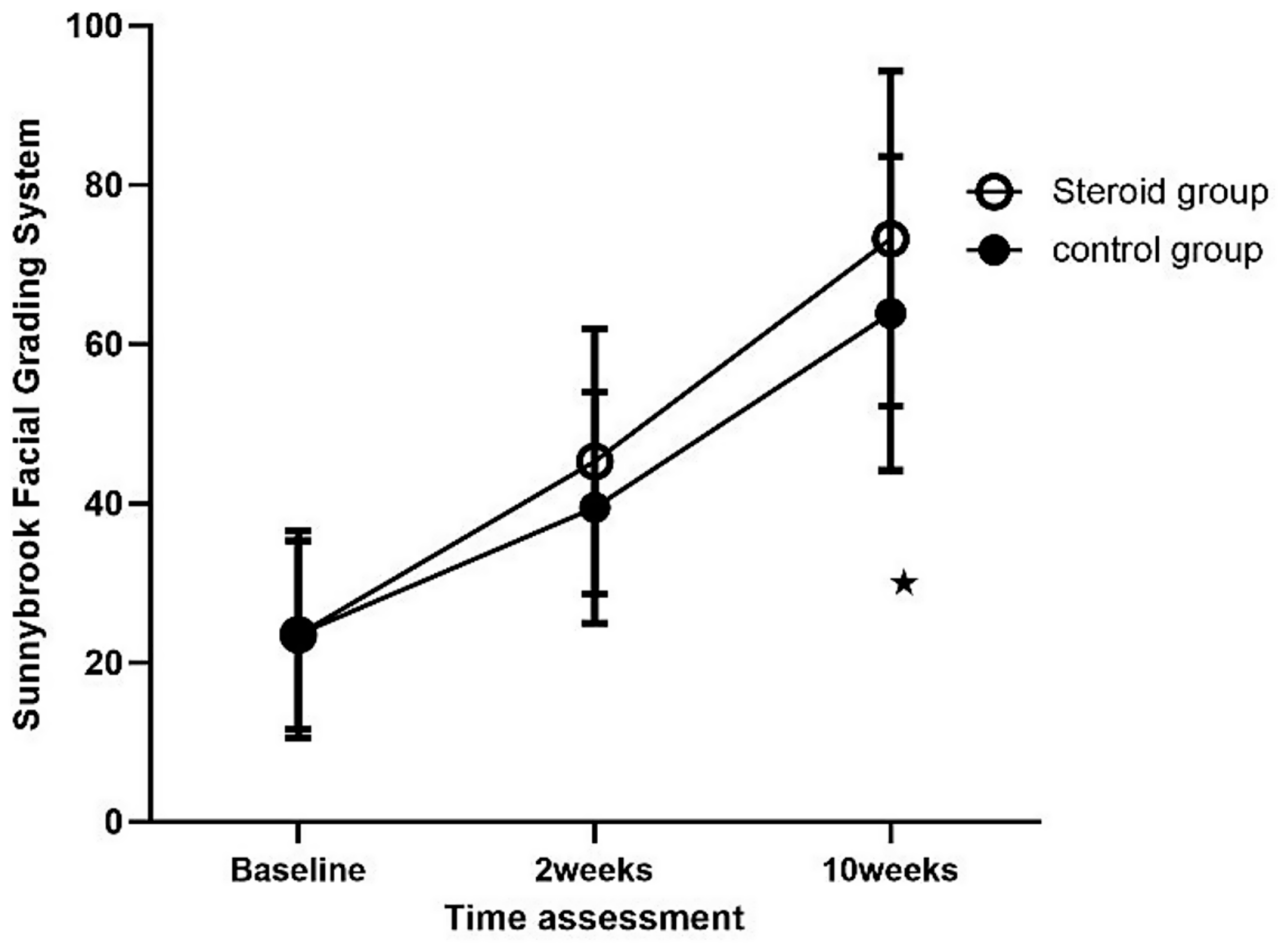
Changes in the mean scores of the Sunnybrook Scale in Bell’s palsy patients between the groups at different time points of assessment.

The Sunnybrook Scale scores of each group were analyzed separately. A non-parametric Friedman test showed that time had a significant effect on both groups (*p* = 0.000 for each group). Mann–Whitney pairwise comparisons were used to compare the overall change in Sunnybrook Scale scores between the two groups at different times of assessment. It showed a significant difference between the two groups with greater improvement in the steroid group at 10 weeks (63.89 ± 19.74 vs. 73.25 ± 21.04, *p* = 0.0021) (Figure 2).

All adverse reactions observed in this study were mild to moderate. In the steroid group, adverse reactions were mainly associated with steroid administration, including: five patients experienced transiently elevated blood glucose, which resolved without intervention; five patients had drug-related gastric discomfort, which was alleviated through dietary adjustments; and three patients reported mild insomnia or a sense of tension. In the control group, two patients developed subcutaneous hematomas after acupuncture but they resolved spontaneously. There were no severe adverse events recorded in either group.

## 5 Discussion

There is strong evidence in favor of the use of steroids as first-line medical conservative treatment for Bell’s palsy (1, 9). The best results of high-dose steroids were achieved when used early in Bell’s palsy (12). Some Bell’s palsy patients without steroids use in the acute phase due to different reasons usually had poor recovery of facial paralysis and sequelae such as facial asymmetry, contracture, and synkinesis. Besides conservative treatment, facial nerve decompression for severe Bell’s palsy was commonly advised within the initial 2 weeks following onset of facial paralysis (13). Nevertheless, other studies have shown that decompression surgery on severe Bell’s palsy patients does not necessarily provide significant improvement in prognosis compared to what can be provided by conservative treatment alone (14). Therefore, at present, decompression surgery for facial paralysis is debatable. For these patients, it still lacked effective treatment protocols. In the present study, we initially observed the effect of steroids on severe refractory Bell’s palsy patients who had a clinical course of 15–30 days and HB grade ≥ IV, and were not given steroids in the acute phase. The results showed that the use of steroids could more effectively facilitate the recovery of severe refractory facial paralysis in the recovery phase.

The cause of Bell’s palsy is not known. The possible etiologic factors may include anatomical causes, viral infection, vascular ischemia, immune-inflammatory diseases, and acute cold exposure (4). Although the pathogenesis of Bell’s palsy has not been fully explained, it is closely associated with inflammation and swelling of facial nerve resulting in its entrapment in the facial canal. Liston and Kleid first demonstrated the histological changes in facial nerve inflammation in Bell’s palsy (15). Yilmaz et al. (16) found that the serum levels of cytokine interleukin-1 (IL-1), IL-6, and tumor necrosis factor-alpha (TNF-α) in patients with Bell’s palsy were much higher. Kinar et al. (17) also discovered that systemic immune-inflammation index and neutrophil-to-lymphocyte ratio, as important inflammatory indicators, significantly increased in patients with Bell’s palsy. Red cell distribution width, another key indicator of inflammation, was found to be significantly increased in patients with Bell’s palsy (18). The rise of these indicators suggested a poor prognosis for Bell’s palsy. A number of studies have shown that most Bell’s palsy patients had edematous swelling of the facial nerve persisting up to 3 months after symptom onset (19, 20). These findings provide further support for the inflammatory pathogenesis of Bell’s palsy.

In clinical, we observed that some of the Bell’s palsy patients had a poor prognosis due to failure to receive steroid therapy in time after the onset of symptoms. These patients with a clinical course of 15–30 days and HB grade ≥ IV were always refractory. Consistent with the previous report (21), our clinical experience has validated that more severe palsy (HB grade ≥ IV) was associated with worse recovery than less severe palsy (HB grade ≤ III). We discovered that facial nerve enhancement MRI in these refractory Bell’s palsy patients revealed varying levels of enhancement in various parts of the facial nerve, indicating that facial nerve swelling and inflammation still present in these patients.

Therefore, this study was aimed at determining the impact of steroids on the prognosis of refractory Bell’s palsy patients with a clinical course of 15–30 days and HB grade ≥ IV, who had not received steroids after the onset of symptoms. The results indicated that the facial nerve function improved significantly when steroid therapy was added to neurotrophic drugs, acupuncture treatment and mirror exercises. Steroid therapy increased the rate of grade I in the HB Grading Scale by almost two times.

In this study, we selected acupuncture as one part of routine treatments. Acupuncture is widely practiced in the treatment of Bell’s palsy in China. It could facilitate nerve regeneration, nerve excitability, and increase muscle contraction, blood circulation (22). Xu et al. (23) demonstrated that acupuncture could facilitate recovery of Bell’s palsy. A clinical practice guideline has given the following “Grade A” recommendation: For Bell’s palsy within 3 months, any one of acupuncture, drugs, or an acupuncture + drug combination is suitable for patients with mild facial palsy; acupuncture or an acupuncture + drug combination is patients is suitable for with severe facial palsy. When durations longer than 3 months, acupuncture is more suitable (23). Although some people hold reservations about the use of electrical stimulation in facial paralysis because they believe it may interfere with the natural regeneration process, recent studies have shown that under appropriate parameters, electro-acupuncture not only can be safely used in non-acute phase patients, but also promote nerve repair through multiple mechanisms (24). Given these, acupuncture was considered necessary in the routine treatments.

This study found that even when the course of the disease has taken 15–30 days, and patients still have inflammation of facial nerves, further application of steroids during the recovery phase can still significantly facilitate the recovery of facial nerve function and increase the rehabilitation rate. This discovery expands the time window in which steroids may be used in treating Bell’s palsy and provides a new therapeutic strategy for clinical practice, which is significantly different from the strategy in current mainstream guidelines that only suggest steroids as an option in the acute stage (within 72 h after onset).

The patients included in this study had a clinical course ranging from 15 to 30 days, which is a relatively wide time window. Although this design reflects the status of refractory patient populations in real-world clinical practice, it may also result in possible heterogeneity among the study subjects. Future prospective studies should aim to narrow this observation window. In future studies, we will further clarify the correlation between the recovery of facial nerve function (assessed through scales) and the facial nerve conduction status (measured through facial nerve electrography) in patients after steroid use.

## 6 Conclusion

This study demonstrates that steroid administration facilitates the recovery of facial nerve function in patients with severe refractory Bell’s palsy (HB grade ≥ IV) during the recovery stage (15–30 days post-onset) who did not receive steroids in the acute stage.

## Data Availability

The raw data supporting the conclusions of this article will be made available by the authors, without undue reservation.
